# MURDEROUS MYTHOMANIA: Psychopathology of lying – Apropos a Clinical Case

**DOI:** 10.1192/j.eurpsy.2022.1829

**Published:** 2022-09-01

**Authors:** S. Jesus, A. Costa, G. Simões, M. Almeida, P. Garrido

**Affiliations:** Baixo Vouga Hospital Centre - EPE, Psychiatry And Mental Health Department, Aveiro, Portugal

**Keywords:** mythomania, personality, Psychopathology, lying

## Abstract

**Introduction:**

The capacity for lying is a common human phenomenon with evolutionary explanations, in which one seeks to deceive usually to avoid harmful or undesired consequences. The spectrum of lies is vast and varies from the content to the motivation. Pathological lying has the potential to affect mental evaluations thus motivating an important discussion regarding this behaviour.

**Objectives:**

The authors aim to explore the psychopathological concept and spectrum of pathological lies, from their underlying motives to their implications and challenges in psychiatric diagnosis with recourse to a clinical case example.

**Methods:**

A review of pertinent literature on the topic with focus on that which is most relevant to the theme was included. The authors present the clinical case of a middle-aged female who presented with mythomania which included the fabrication of having attempted murder.

**Results:**

The literature demonstrates a relationship between compulsive lying and personality disorders. Head trauma and other central nervous system issues may also play a role. Some traits may facilitate the detection of deception, such as dramatic and unmotivated constructs with a positive self-portrayal. The clinical case description correlates the personality factors associated with mythomania, namely antisocial personality disorder, differing from the typical presentation as her fabrications portrayed her negatively.

**Conclusions:**

The implication of pathological lying is that it may interfere with mental assessment thus altering, by way of deception, the psychiatric evaluation as lies may be difficult to detect upon a first evaluation. The psychiatrist should be alerted to the possibility of fabrication when dealing with a patient with predisposing factors.

**Disclosure:**

No significant relationships.

